# Leadership behaviour, team effectiveness, technological flexibility, work engagement and performance during COVID-19 lockdown: An exploratory study

**DOI:** 10.4102/sajip.v47i0.1829

**Published:** 2021-04-21

**Authors:** Lome Koekemoer, Leon T. de Beer, Karissa Govender, Marissa Brouwers

**Affiliations:** 1 Yellow Seed Consulting, Johannesburg, South Africa; 2 Society for Industrial and Organisational Psychology of South Africa (SIOPSA), Pretoria, South Africa; 3 WorkWell Research Unit, Faculty of Economic and Management Sciences, North-West University, Potchefstroom, South Africa; 4 School of Industrial Psychology and Human Resource Management, Faculty of Economic and Management Sciences, North-West University, Potchefstroom, South Africa

**Keywords:** leadership behaviour, team effectiveness, technological flexibility, work engagement, ‘hard lockdown’

## Abstract

**Orientation:**

The coronavirus disease 2019 (COVID-19) pandemic has taken the world by storm. Little is known about leadership, motivation and employee performance during pandemics and associated lockdowns.

**Research purpose:**

The current study investigated a model of leadership behaviour, team effectiveness, technological flexibility, work engagement and performance in the context of a ‘hard lockdown’ in South Africa.

**Motivation for the study:**

As a result of the COVID-19 pandemic and the resulting lockdown, it was considered from an academic-practitioner perspective to explore leadership behaviour, team effectiveness, technological flexibility, work engagement and performance.

**Research approach/design and method:**

Specifically, remote workers were sampled online via social media (*n* = 229). Structural equation modelling methods were used to analyse the data, also controlling optimism and pessimism at the item level.

**Main findings:**

The results showed that the resources of leadership behaviour and team effectiveness had direct positive paths to work engagement and that work engagement had a positive path to two performance factors: adaptivity and proactivity. Furthermore, there were significant indirect relationship present from leadership behaviour and team effectiveness to both adaptability and proactivity through work engagement.

**Practical/managerial implications:**

From the evidence it seems appropriate to recommend that organisations explore fostering the employee job resources in order to positively impact work engagement, which in turn can have beneficial performance outcomes for organisations who have employees working remotely whilst the COVID-19 regulations remain in force.

**Contribution/value-add:**

This study was unique as it sampled from employees ‘locked down’ during a pandemic and gauged their perceptions of leadership behaviour, team effectiveness, technological flexibility, work engagement and performance.

## Introduction

There is no doubt that coronavirus disease 2019 (COVID-19), and the associated lockdown strategies instituted by most governments, have been the most significant events of the 21st century. Governments around the world have taken various approaches to ‘locking down’ citizens, employees, businesses, other organisations and local and international travel to curb the spread of the virus and to ‘flatten the curve’ of the infection rate in their populations (see Thunstrom, Newbold, Finnoff, Ashworth, & Shogren, 2020). The South African government declared a national state of disaster in accordance with law (Disaster Management Act, 2002) and has taken a ‘hard lockdown’ strategy, which indicates not only a *very* restrictive set of regulations but also arguably one of the most restrictive sets of lockdown regulations in the world when compared to Australia and the United Kingdom (Writer, 2020). The lockdown in South Africa started on 26 March 2020, and was initially meant to last for 3 weeks (Ramaphosa, 2020a). The lockdown was then extended by another 2 weeks (Ramaphosa, 2020b), with a new five-phase lockdown system taking over from the 5-week lockdown on 01 May 2020 – somewhat fittingly, on International Labour Day. However, little is known about the impact that the extended lockdown has on employees who are now working from home away from their usual office habitat. More specifically, we have little knowledge of how the current situation impacts employee performance and the mechanisms through which performance is impacted.

During the hard lockdown, only occupations and organisations classified as essential service workers are allowed out of their homes to go to work whilst all other business premises remain closed (Government of South Africa, 2020). However, employees in other sectors could work from home and many ongoing innovations have been implemented in order to make this possible. This situation poses a challenge to leadership, and little research exists that support the effect of leadership on work engagement and performance on remote workers during a lockdown scenario. Given the unprecedented situation, it is safe to say that leadership will remain one of the most important determinants of the successful navigation of outcomes for organisations (Lacerda, 2019), as leaders are navigating these conditions of uncertainty, complexity and volatility not seen since the Great Recession of 2008 (Walker, Earnhardt, Newcomer, Marion, & Tomlinson, 2016). The decision-making of leaders surrounding technologies made available to staff/clients will not only impact team effectiveness in a remote work situation but also quite possibly determine the longevity of the organisation.

Recently, a myriad of online research arose, from various spheres of interest, assessing the impact of COVID-19 and lockdowns on *inter alia* the stress and health of employees or citizens in general (e.g. Brown, Doom, Lechuga-Peña, Watamura, & Koppels, 2020). Although the results of these surveys are valuable, they tend to focus on the negative aspects. This can be problematic as it may inadvertently add to potential stress levels by unnecessarily focusing on job insecurity and similar constructs, depending what is asked and how it is phrased. Consequently, the researchers decided to take a more positive psychological approach in the current study. We did so by focusing on specific resources that may be important to employees at home, given the circumstances of the lockdown, and that play a motivational role in enhancing performance. Therefore, the general objective of this study was to explore a motivation model of performance enhancement amongst a sample of South African employees working from home during the government-enforced lockdown.

## Theoretical foundation

As theoretical framework, the study draws on the motivational process of the job demands-resources (JD-R) model (Bakker & Demerouti, 2017). Specifically, the motivational process of the JD-R model explains that work engagement (comprising vigour, dedication and absorption) results because of job resources provided or available to employees within their work environment and that this work engagement, in turn, has an impact on employee outcomes (Lesener et al., 2019; Schaufeli, 2017). In the current study, performance (proficiency, adaptivity and proactivity) is the outcome measure. Therefore, job resources are associated with optimal psychological functioning (Van den Broeck, De Cuyper, De Witte, & Vansteenkiste, 2010) and favourable organisational outcomes in general.

### Job resources, work engagement and performance

As mentioned previously, job resources lead to work engagement and positive organisational outcomes (Bakker & Demerouti, 2017). Demerouti, Bakker, Nachreiner and Schaufeli (2001) describe job resources as the organisational aspects of the job that are instrumental in achieving work goals and may also reduce job demands. The resources used in this study as determinants of work engagement and performance are leadership behaviour (as a supervisory support resource), team effectiveness (as a social/collegial support resource) and technology and flexibility (as an organisational support resource).

Leadership behaviour was used in this study to gauge the perceptions of the participants of their leaders in response to the change that was necessary because of the lockdown. Specifically, this was based on work by Prewitt and Weil (2014) where they explain that leadership can change organisational stress using technical (quick solutions, setting procedures for known problems) or adaptive techniques (addresses fundamentals and dysfunctional dynamics), but crisis leadership realises that this is insufficient in the long-term without an adaptive response. Leadership has been shown to impact motivational states such as work engagement and outcomes such as performance (Caulfield & Senger, 2017; Stander, De Beer, & Stander, 2015). In this study, performance is looked at from the perspective of the positive work role behaviours described by Griffin, Neal and Parker (2007); specifically, proficiency (fulfils the prescribed or predictable requirements of the role), adaptivity (copes with, responds to and supports change) and proactivity (initiates change, is self-starting and future directed; Griffin et al., 2007). Work engagement has also been shown to affect performance (Gauche & De Beer, 2018).

Team effectiveness was a collegial resource used in this study and indicates support from colleagues in work – in addition to considering the effectiveness of the teams in reaching work goals. De Beer, Rothmann and Pienaar (2012) found job resources that contained colleague support as a positive predictor of work engagement and buffer for burnout. In terms of the resource technological flexibility which indicates support from organisations to workers in order to have remote working arrangements or be able to work remotely, Felstead and Henseke (2017) found that detachment of work was a growing trend, and that remote working is related to increased job satisfaction and work-related well-being, but that those advantages evoke work intensification and an increased inability to switch off.

Therefore, the stated exploratory model indicates a potential mediating effect of work engagement in the relationship between the antecedents (leadership, behaviour, team effectiveness and technological flexibility) and the performance variables (Zhao, Lynch, & Chen, 2010). Indeed, research has shown work engagement to be an important mediating variable in this regard (cf. Bakker & Demerouti, 2017; De Beer et al., 2012).

## Method

### Research participants

A cross-sectional quantitative survey design was used, and the sample consisted of 229 participants (*n* = 229). Although the survey is open for the duration of the lockdown, the data from 16 April up to 26 April 2020 was used for this study. Therefore, at that time, the lockdown was experienced for a total of 20 days (about 1 work month). Specifically, an online survey was created with a free licence account on the *QuestionPro* platform and shared on social media outlets (i.e. Facebook and LinkedIn) by *inter alia* the Society of Industrial and Organisational Psychology of South Africa. Therefore, participants were completely free to decide whether they would like to participate in the study or not, as such this sample is a non-probability sample. The inclusion criterion for the study was that participants needed to be working during the lockdown.

The mean age of the participants was 38.08 years (standard deviation [SD] = 10.11) and the majority identified as female (*n* = 159; 69.43%) and the rest identified as male. Furthermore, 68 participants (29.69%) indicated that they were employees who could be classified as essential service workers and 111 (48.47%) indicated that they had dependent children at home or dependents older than 60 (*n* = 30; 13.10%). In terms of relationship status, 122 (55.02%) participants indicated that they were married.

### Measuring instruments

The researchers considered carefully the wording of the survey instructions to avoid any unnecessary words such as ‘crisis’ to avoid influencing the mind frame of employees but attempted to be as neutral as possible. Furthermore, all scales were adapted in such a manner that the participants would answer thinking about the last few weeks of lockdown. This entailed an instruction in some instances (e.g. ‘During the last few weeks’) and changes in tenses to suit the above adaption.

*Leadership behaviour* was measured with a 9-item scale created by the researchers using the work of Prewitt and Weil (2014) as a guideline on a scale from 1 (strongly disagree) to 5 (strongly agree). This scale measured the perceptions of participants regarding their leader’s behaviour during the past few weeks (see Table 1). An example item from this scale was ‘During the last few weeks my manager… demonstrated courage and active responsibility for dealing with the change’. Cronbach’s a coefficient for this scale was 0.94 and, therefore, considered highly acceptable.

**TABLE 1:** Standardised loadings for the latent factors from the confirmatory factor analysis model.

Factor	Item	Loading	SE	*p*
Leadership behaviour	*During the last few weeks, my manager …*			
	1. Remained poised and calm in dealing with the change	0.63	0.07	0.001
	2. Demonstrated courage and active responsibility for dealing with the change	0.72	0.06	0.001
	3. Demonstrated a personal investment in the team	0.80	0.03	0.001
	4. Encouraged employees to remember the team’s core purpose and goals	0.72	0.05	0.001
	5. Led by example in accordance with our organisational values	0.84	0.04	0.001
	6. Promoted team cohesiveness and collaboration amongst our team	0.82	0.03	0.001
	7. Provided a clear plan to follow during the change	0.76	0.04	0.001
	8. Provided an opportunity for team members to give their inputs, feedback and ideas	0.75	0.04	0.001
	9. Empathised with the stress and strain employees were experiencing	0.76	0.04	0.001
Team effectiveness	*During the last few weeks, team members in my team …*			
	1. Displayed high levels of trust and helped each other	0.60	0.07	0.001
	2. Confronted problems head-on and resolved it quickly	0.83	0.03	0.001
	3. Displayed high levels of commitment towards achieving the defined team goals	0.87	0.04	0.001
	4. Accepted personal accountability to deliver their share of work to achieve the defined team goal	0.85	0.04	0.001
	5. Collaborated to achieve good results			
Technological flexibility	*During the last few weeks, …*			
	1. My organisation’s technology infrastructure enables remote and flexible working	0.82	0.06	0.001
	2. My organisation was ready to go into remote working in a crisis situation	0.77	0.04	0.001
	3. My organisation has enabled me to work remotely successfully	0.89	0.04	0.001
Work engagement	*Thinking about the last few weeks, …*			
	1. When working, I felt bursting with energy	0.81	0.06	0.001
	2. I am enthusiastic about my job	0.79	0.05	0.001
	3. When I worked, I was immersed in my work	0.65	0.08	0.001
Performance: Proficiency	*Over the last few weeks, you …*			
	1. Carried out the core parts of your job well	0.77	0.06	0.001
	2. Provided help to co-workers when asked, or needed	0.66	0.07	0.001
	3. Presented a positive image of the organisation to other people (e.g. clients)	0.51	0.08	0.001
Performance: Adaptivity	1. Coped well with changes that impacted your core tasks	0.78	0.06	0.001
	2. Responded constructively to changes in the way your team works	0.81	0.04	0.001
	3. Quickly accepted changes implemented in the way the organisation operates	0.76	0.05	0.001
Performance: Proactivity	1. Came up with ideas to improve the way in which your core tasks are done	0.75	0.06	0.001
	2. Developed new and improved methods to help your work unit perform better	0.84	0.06	0.001
	3. Involved yourself in changes that are helping to improve the overall effectiveness of the organisation	0.60	0.06	0.001

Loading, standardised factor loading; SE, standard error.

All *p* values < 0.001.

*Team effectiveness* was measured with a 5-item scale which was developed by the researchers using Patrick Lencioni’s Team Effectiveness model as the guideline (cf. Hamlin, 2008). The scale ranged from 1 (strongly disagree) to 5 (strongly agree). An example item from this scale was ‘During the last few weeks my team members … accepted personal accountability to deliver their share of work to achieve the defined team goal’. Cronbach’s a coefficient for this scale was 0.89 and therefore considered acceptable.

*Technological flexibility* was measured with a 3-item scale developed by the researchers, ranging from 1 (strongly disagree) to 5 (strongly agree). An example item from this scale was ‘My organisation’s technology infrastructure enables remote and flexible working’. Cronbach’s a coefficient for this scale was 0.88, which is also highly acceptable.

*Work engagement* was measured with the 3-item ultrashort Utrecht Work Engagement Scale (Schaufeli, Shimazu, Hakanen, Salanova, & De Witte, 2019) on a scale of 1 (never) to 7 (always). An example item is ‘When working, I felt bursting with energy’. Cronbach’s a coefficient for this scale was 0.82, which is acceptable.

*Performance* was measured with an adapted and shortened 9-item version of the Work Role Performance scale (Griffin et al., 2007). For each component, one item was used for the individual, team and organisation, respectively, on a scale from 1 (strongly disagree) to 5 (strongly agree) and therefore consisted of three items for each of the three components: (1) proficiency (α = 0.72; e.g. ‘Over the last few weeks, you… carried out the core parts of your job well’), (2) adaptivity (α = 0.87; e.g. ‘Over the last few weeks, you…actively focused on learning new skills to better deal with the change at hand’) and (3) proactivity (α = 0.82; e.g. ‘Over the last few weeks, you…came up with ideas to improve the way in which your core tasks are done’).

Finally, two single-item non-demographic control questions were asked for use in the analysis about the participant’s general levels of *optimism* and *pessimism* from the Scale Optimism–Pessimism-2 (SOP2; Kemper, Wassermann, Hoppe, Beierlein, & Rammstedt, 2017) on a scale of 1 (not at all pessimistic/optimistic) to 7 (very optimistic/pessimistic). In the current study, the items were used individually as research has shown that optimism and pessimism are not necessarily perceived on a continuum (see Hummer, Dember, Melton, & Schefft, 1992).

### Statistical analyses

**FIGURE 1:**
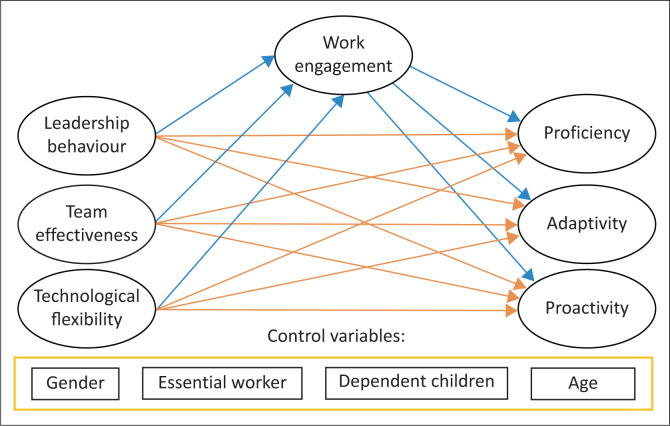
The conceptual structural model.

With Mplus 8.4 (Muthén & Muthén, 2019), confirmatory factor analysis (CFA) was applied with the robust maximum likelihood estimation method – which is robust against the possibility of non-normality in the data. Importantly, given the context in which data collection was done and the potential biases of participants in answering the survey, all the items were regressed on the observed scores for optimism and pessimism to lessen any potential biases. Figure 1 is the conceptual CFA model which was provided by Mplus. Thus, in the CFA model controls were estimated at the item level in creation of the measurement model. Standard fit measures were considered for the models: comparative fit index (CFI), Tucker–Lewis index (TLI), root mean square error of approximation (RMSEA) and the standardised root mean square residual (SRMR).

Furthermore, correlations were also considered amongst the classic guidelines of 0.30 and above for a medium effect size and 0.50 and above for a large effect size.

For the structural model, additional demographic control variables were added to the model in *which all the latent variables were regressed* on gender, essential services worker, and age, seeing whether these three demographic variables were statistically significant predictors of the variables, thereby also controlling for these variables in explaining variance. Essential service worker in this context indicated whether someone was an essential service worker as defined by the government but working from home. The focus was on the direction of the standardised b coefficients and the statistical significance associated with each of the parameters, which is set as a *p* < 0.05. For all potential indirect effects in the model, bootstrap resampling was used (10 000 draws), paying attention to the 95% confidence intervals (CI) of the estimates in order to ascertain whether the estimate crossed zero (Hayes, 2017).

## Results

### Model fit: confirmatory factor analysis model and control variables

The CFA showed that the measurement model (see Figure 1) was an acceptable fit to the data: CFI = 0.92; TLI = 0.90; RMSEA = 0.058; SRMR = 0.054. As can be seen in Table 3 all the factor loadings were statistically significant and acceptable. Moreover, the standard errors were relatively small, indicating accuracy of the estimation process.

Regarding the control variables of optimism and pessimism at item level, the following was evident: Optimism explained small, but statistically significant, variance in all of the leadership behaviour items except for item 2. For team effectiveness, pessimism had a statistically significant negative relationship with item 1. No statistically significant relationships were observed with technological flexibility’s items (*p* > 0.05). Optimism, but not pessimism, had a statistically significant with all of the work engagement items. In terms of the performance items, optimism had av statistically significant relationship with the items of proficiency (items 1 and 3), adaptivity (item 1) and proactivity (items 1 and 2). Finally, pessimism had statistically significant negative relationships with the items of adaptability (items 1 and 3). Interestingly, both optimism and pessimism had an effect on item 1 of adaptability. The reader can refer to Table 1 below to read the item text for each item corresponding to the specific number.

### Correlations

The correlations (see Table 2) showed that all the significant relationships between variables were positive and that most of the effect sizes could be described as medium. Specifically, leadership behaviour had a positive correlation with all of the variables with medium effect but not with proactivity (*r* = 0.21; small effect). The relationship between technological flexibility and proactivity was not statistically significant. The highest correlation was between proficiency and adaptivity (*r* = 0.82; large effect).

**TABLE 2:** Correlation matrix for the latent variables.

Variables	1	2	3	4	5	6	7
1. Leadership behaviour	0.94	-	-	-	-	-	-
2. Team effectiveness	0.44†	0.89	-	-	-	-	-
3. Technological flexibility	0.42†	0.26	0.88	-	-	-	-
4. Work engagement	0.45†	0.38†	0.21	0.82	-	-	-
5. Performance: Proficiency	0.31†	0.26	0.34†	0.30†	0.72	-	-
6. Performance: Adaptivity	0.35†	0.29	0.38†	0.38†	0.82‡	0.87	-
7. Performance: Proactivity	0.21	0.14	−0.03	0.35†	0.20	0.36†	0.82

Note: Cronbach’s reliability coefficients in brackets on the diagonal; all correlations statistically significant *p* < 0.001.

† , Medium practical effect.

‡ , Large practical effect.

### Structural paths

**FIGURE 2:**
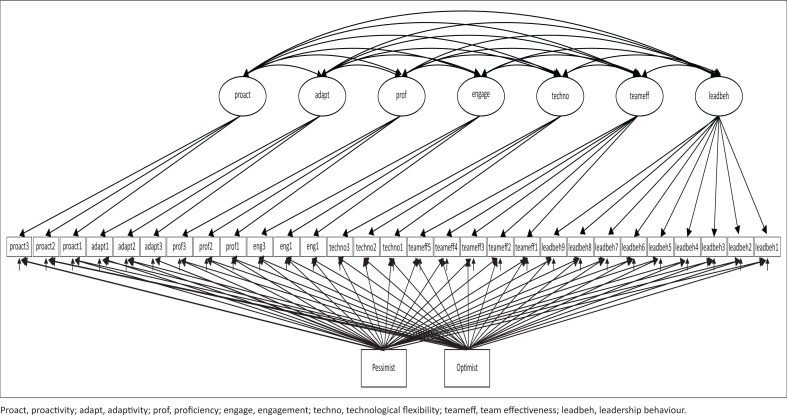
The confirmatory factor analysis model controlling for optimism and pessimism at the item level.

In line with the conceptual model in Figure 2, a structural model was created. The fit of this model was adequate: CFI = 0.92; TLI = 0.89; RMSEA = 0.056; SRMR = 0.062. The results are given in Table 3.

**TABLE 3:** Path results for the structural model.

Structural path	β	SE	*p*	Result
Leadership behaviour → Work engagement	0.31	0.09	0.001	Significant
Leadership behaviour → Proficiency	0.09	0.14	0.523	Not significant
Leadership behaviour → Adaptivity	0.08	0.13	0.537	Not significant
Leadership behaviour → Proactivity	0.12	0.13	0.349	Not significant
Team effectiveness → Work engagement	0.24	0.08	0.003	Significant
Team effectiveness → Proficiency	0.10	0.12	0.409	Not significant
Team effectiveness → Adaptivity	0.10	0.10	302	Not significant
Team effectiveness → Proactivity	0.01	0.10	0.956	Not significant
Technological flexibility → Work engagement	0.05	0.13	0.719	Not significant
Technological flexibility → Proficiency	0.22	0.13	0.097	Not significant
Technological flexibility → Adaptivity	0.26	0.12	0.027	Significant
Technological flexibility → Proactivity	−0.14	0.09	0.128	Not significant
Work engagement → Proficiency	0.21	0.16	0.201	Not significant
Work engagement → Adaptivity	0.27	0.13	0.031	Significant
Work engagement → Proactivity	0.33	0.10	0.002	Significant

*B*, Standardised b coefficient; SE, standard error; *p*, two-tailed statistical significance.

As can be seen, leadership behaviour predicted work engagement positively (β = 0.31; SE = 0.09; *p* < 0.001) but did not have any other direct relationship in the sample. Similarly, technological team effectiveness also predicted work engagement positively (β = 0.24; SE = 0.08; *p* = 0.003). Of the resources, technological flexibility was the only to predict a performance variable, specifically adaptivity (β = 0.26; SE = 0.12; *p* = 0.027). Lastly, work engagement did not predict proficiency (*p* = 0.201) but did predict adaptivity (β = 0.27; SE = 0.13; *p* = 0.031) and proactivity (β = 0.33; SE = 0.10; *p* = 0.002).

In terms of the control variables’ structural paths to all the factors, the overwhelming majority of the results were non-significant except for the positive relationship of age on work engagement – indicating that the higher the age of the participant, the higher the work engagement (β = 0.17; SE = 0.07; *p* = 0.014), but a negative relationship for age on technological flexibility (β = -0.33; SE = 0.10; *p* = 0.002) – indicating that as age increased the technological flexibility decreased. Furthermore, being male had a significant relationship with technological flexibility in the sample. Lastly, being a remote essential service worker had a significant positive relationship with proactivity (β = 0.14; SE = 0.07; *p* = 0.035).

### Indirect relationships (mediation)

Results from the bootstrapped resampling showed that there were four noteworthy indirect relationships in the model. The first two relationships were leadership behaviour to adaptability through work engagement (estimate = 0.11; 95% CI [0.03, 0.21]) and team effectiveness to adaptability through work engagement (estimate = 0.07; 95% CI [0.02, 0.15]). Similarly, in the final two relationships both of these indirect relationships also existed for proactivity, that is leadership behaviour (estimate = 0.15; 95% CI [0.08, 0.24]) and team effectiveness (estimate = 0.09; 95% CI [0.04, 0.17]) to proactivity through work engagement.

## Discussion

The aim of this study was to explore the relationship of leadership behaviour, team effectiveness, technological flexibility, work engagement and performance within the context of the COVID-19 lockdown in South Africa. The sample comprised employees working remotely.

Results indicated that leadership behaviour and team effectiveness were important structural paths for work engagement but not technological flexibility – even though one should consider the correlation was significant and positive. Leadership behaviour is a type of supervisory support resource, and team effectiveness has been argued as a collegial resource, both forming what has been coined as social support as it involves the supervisor and colleagues the employee works with. This, of course, is enshrined within the JD-R model, but social exchange theory provides an avenue of explanation in that it presents that social behaviour is the resultant of an exchange process with the intention to maximise benefits and minimise costs (cf. Cropanzano et al., 2017).

Technological flexibility had a positive structural path to the adaptivity performance component. Considering the current situation in which the employees find themselves, it makes sense that the degree to which technological flexibility is provided by the organisation, that is, the level of remote working implemented has a significant impact on how employees adapt to and cope with the changes necessarily in order to successfully work from home. Indeed, Beauregard, Basile and Canónico (2019) suggest organisations implement evidence-based practices in order to prepare and manage employees’ remote working. Therefore, it is important for organisations to not only enable employees to work remotely, but that this is done in an evidence-backed manner from the available body of literature.

In terms of the performance factors and work engagement, work engagement was a positive predictor of both adaptivity and proactivity. Given the context of COVID-19 and the rapid changes that must be considered and implemented for the organisation to survive, the positive effect of work engagement on performance cannot be discounted. This is in line with broaden-and-build theory that indicates that upward spirals of positive emotions obviate downward spirals and lead to positive outcomes (Vacharkulksemsuk & Fredrickson, 2013).

Finally, even though leadership behaviour and team effectiveness did not have direct relationships to performance in the form of adaptivity and proactivity, there were significant indirect relationships from those variables to these performance variables through work engagement. This indicates the importance of work engagement in these relationships and can be described as an indirect-only effect, that is, the relationship would not be present without work engagement (Zhao et al., 2010). The results are in line with the studies that have shown work engagement to be an important mediator in the relationships between antecedents and individual and organisational outcomes (e.g. Bakker & Demerouti, 2017; De Beer et al., 2012).

Therefore, the recommendation of this study to organisations and their leadership is to foster work engagement by providing employees with the needed job resources during this difficult time and that in turn will be correlated with the positive performance outcomes.

## Limitations and recommendations

The convenience sampling of the study necessitates that we acknowledge that the external validity of the results should be cautioned, also given the unique context of the study being conducted in the Republic of South Africa. However, representativity in the current lockdown situation is hard to gauge and it would have been extremely difficult to collect a representative sample as no sampling frame is available. Furthermore, given the inequality present in South Africa, this sample excludes blue-collar workers who were not working from home and who do not have Internet access in rural area. However, given the lockdown regulations, it is practically impossible to gain access to those employees and we had to settle for people who wanted to voluntarily participate.

A second limitation worth noting is that we hoped for a larger sample size. When modelling, more data are always better; however, when the sum scores of the variables are created as opposed to the item-level approach used here, the observed sum score-based model also shows the same statistically significant results, indicating that the more complex measurement model did not impact the results. Additionally, because of the pragmatic approach taken, the researchers created/adapted scales, and these scales worked well, but the validity of these scales should further be investigated in the future studies. Specifically in terms of the SOP2, we wanted to ensure that we control for the effects of both these components in this study, given that the SOP2 has not been formally validated in English even though the face validity of the questions seem straightforward and that it has been suggested as a tool to use because of its cross-cultural evidence (Kemper et al., 2017).

This study also demonstrated the importance of controlling for potential bias in the answering of items, specifically by considering both optimism and pessimism scores and the effect this might have at the item level. Researchers in the field of the social sciences should, therefore, consider including similar control variables in their studies to ensure that the modelling of the relationships between variables in the structural model is accurate.

Future studies can also take a person-centred approach which would allow for the creation of profile categories based on items or factor scores of the latent variables. As a final, but no less important recommendation, research on the psycho-social impact of the COVID-19 pandemic and residual effects of the lockdown on the employed and unemployed should be done to obviate the potential lasting effects as effectively and efficiently as possible.
